# Tylvalosin tartrate inhibits PRRSV replication by suppressing cellular pyroptosis through the TLR4/NF-κB signaling pathway

**DOI:** 10.1128/jvi.00202-26

**Published:** 2026-04-30

**Authors:** Shi-qin Zhang, Xin-lei Li, Cheng Yang, Dao-wen Li, Xiao-hong Deng, Wei Hu, Xing-yao Pei, Wen-juan Zhang, Xiao-xue Yu, Ying-Feng Sun

**Affiliations:** 1 College of Animal Science and Veterinary Medicine, Tianjin Agricultural University 91633, Tianjin, China; 2 College of Veterinary Medicine, China Agricultural University 630101 , Beijing, China; 3 College of Veterinary Medicine, Xichang University 381931, Xichang, China; 4 College of Veterinary Medicine, Gansu Agricultural University 739715, Lanzhou, China; Loyola University Chicago - Health Sciences Campus, Maywood, Illinois, USA

**Keywords:** tylvalosin tartrate, TLR4, pyroptosis, PRRSV, replication

## Abstract

Tylvalosin tartrate, a potent antimicrobial agent, is clinically observed to mitigate porcine reproductive and respiratory syndrome virus (PRRSV) infection, yet its mechanism remains unclear. Here, we demonstrate that tylvalosin tartrate treatment significantly reduces serum viremia and inflammatory cytokine production, correlating with symptom alleviation in piglets infected with lineage 1 (L1) PRRSV. *In vitro*, it effectively inhibits the replication of not only L1 PRRSV but also other major lineage strains (L8.1/CH-1a-like, L5/VR2332-like, and L8.3/JXA1-like) in porcine alveolar macrophages (PAMs). Transcriptomic analysis suggested that tylvalosin tartrate suppresses PRRSV replication by inhibiting cellular pyroptosis. We confirmed that L1 PRRSV infection induces pyroptosis in PAMs, and viral replication was hindered upon inhibition of caspase-1 (CASP1) or gasdermin D (GSDMD). Tylvalosin tartrate inhibited GSDMD-mediated pyroptosis induced by either L1 PRRSV or lipopolysaccharide (LPS)/nigericin. Further investigation revealed that tylvalosin tartrate acts upstream by suppressing the activation of the TLR4/NF-κB signaling pathway, a key driver of inflammatory gene expression and pyroptosis. Pharmacological inhibition of TLR4 using resatorvid and genetic knockdown through siRNA replicated the antiviral and anti-pyroptotic effects of tylvalosin tartrate. Our findings elucidate a host-directed mechanism by which tylvalosin tartrate restricts PRRSV replication across diverse lineages, highlighting its potential as a multifaceted therapeutic agent against PRRSV infection.

**IMPORTANCE:**

Porcine reproductive and respiratory syndrome (PRRS) causes enormous economic losses worldwide. Controlling PRRSV is challenging due to viral genetic diversity, limited vaccine efficacy, and the lack of specific antiviral drugs. Tylvalosin tartrate, a widely used veterinary antibiotic for respiratory and enteric diseases, has been empirically observed to improve outcomes in PRRSV-affected herds. This study provides novel mechanistic evidence that tylvalosin tartrate possesses direct, broad-spectrum antiviral activity against PRRSV by targeting a host pathway. We show that it inhibits viral replication across major epidemic lineages by dampening the TLR4/NF-κB signaling axis and the subsequent gasdermin D (GSDMD)-dependent pyroptosis. This “old drug, new use” strategy repurposes an approved, safe compound to counteract both viral replication and excessive inflammation—a hallmark of severe PRRS. Our work not only clarifies the molecular basis for its clinical utility but also positions tylvalosin tartrate as a promising host-targeted antiviral and immunomodulatory agent for PRRS management, offering a practical and immediate intervention strategy for the swine industry.

## INTRODUCTION

Porcine reproductive and respiratory syndrome virus (PRRSV) is a globally prevalent pathogen causing significant economic losses in the swine industry (1). Belonging to the *Arteriviridae* family, it is an enveloped RNA virus with a genome of approximately 15 kb (2). Two genotypes exist: PRRSV-1 (European) and PRRSV-2 (North American), with PRRSV-2 being prevalent in China (3). Based on ORF5 gene sequences, PRRSV-2 strains are classified into multiple lineages; lineages 1, 3, 5, and 8 are the most predominant in China, often co-circulating and complicating control efforts (4). Among these, lineage 1 (L1) strains are currently primary epidemic strains (5, 6), while existing vaccines show limited efficacy against the diverse circulating strains (7). Therefore, developing broad-acting antiviral agents is of great importance.

Virus-host interactions crucially determine replication efficiency. PRRSV infection induces severe inflammation, promoting replication and pathogenesis (8, 9). It triggers interleukin-1β (IL-1β) production, linked to reproductive failure (10), and activates the TLR4/NF-κB pathway, which is required for replication (11–13). Similar pro-inflammatory mechanisms involving TLR4/NF-κB activation have been reported for other viruses: influenza A virus (IAV) activates the expression of the TLR4/NF-κB signaling pathway, and the traditional Chinese medicine Liushenwan could inhibit IAV replication by inhibiting the expression of TLR4 and phosphorylated NF-κB both *in vivo* and *in vitro* (14). Similarly, dihydromyricetin inhibits African swine fever virus (ASFV) replication by downregulating TLR4-dependent pyroptosis pathways *in vitro* (15). These findings highlight that TLR4 could serve as a potential target for therapeutic intervention against viral infections.

The NLRP3 inflammasome senses diverse stimuli and, upon activation, cleaves pro-IL-1β and gasdermin D (GSDMD) via caspase-1 (CASP1), leading to IL-1β maturation and GSDMD-mediated pyroptosis (16–19). Viral infections like bovine viral diarrhea virus (BVDV) activate NLRP3 and pyroptosis via NF-κB (20). Recent studies indicate that PRRSV also induces GSDMD-driven pyroptosis in porcine alveolar macrophages (PAMs) via NLRP3 activation (21). However, the link between TLR4/NF-κB and pyroptosis during PRRSV infection is not fully understood.

Tylvalosin tartrate, a third-generation veterinary macrolide antibiotic, is effective against bacterial and mycoplasmal infections (22, 23) and exhibits anti-inflammatory properties by inhibiting NF-κB (24). It also shows antiviral activity against PRRSV *in vitro* (25, 26), and field observations suggest that it can improve outcomes in PRRSV-affected herds (12, 27, 28). However, its mechanism remains elusive. This study demonstrates that tylvalosin tartrate alleviates clinical symptoms, reduces viremia and cytokines in L1 PRRSV-infected piglets, and significantly inhibits the replication of multiple PRRSV lineages (lineage 1/NADC30-like, lineage 8.1/CH-1a-like, lineage 5/VR2332-like, and lineage 8.3/JXA1-like) at a concentration of 3.125 µM in PAMs. We further mechanistically demonstrate that tylvalosin tartrate suppresses PRRSV replication by inhibiting cellular pyroptosis via the TLR4/NF-κB pathway. Given its clinical use, our findings provide novel insights into its broad antiviral mechanism and potential strategies for PRRSV control.

## RESULTS

### Tylvalosin tartrate alleviates clinical symptoms and suppresses replication in L1 PRRSV-infected piglets

**Fig 1:**
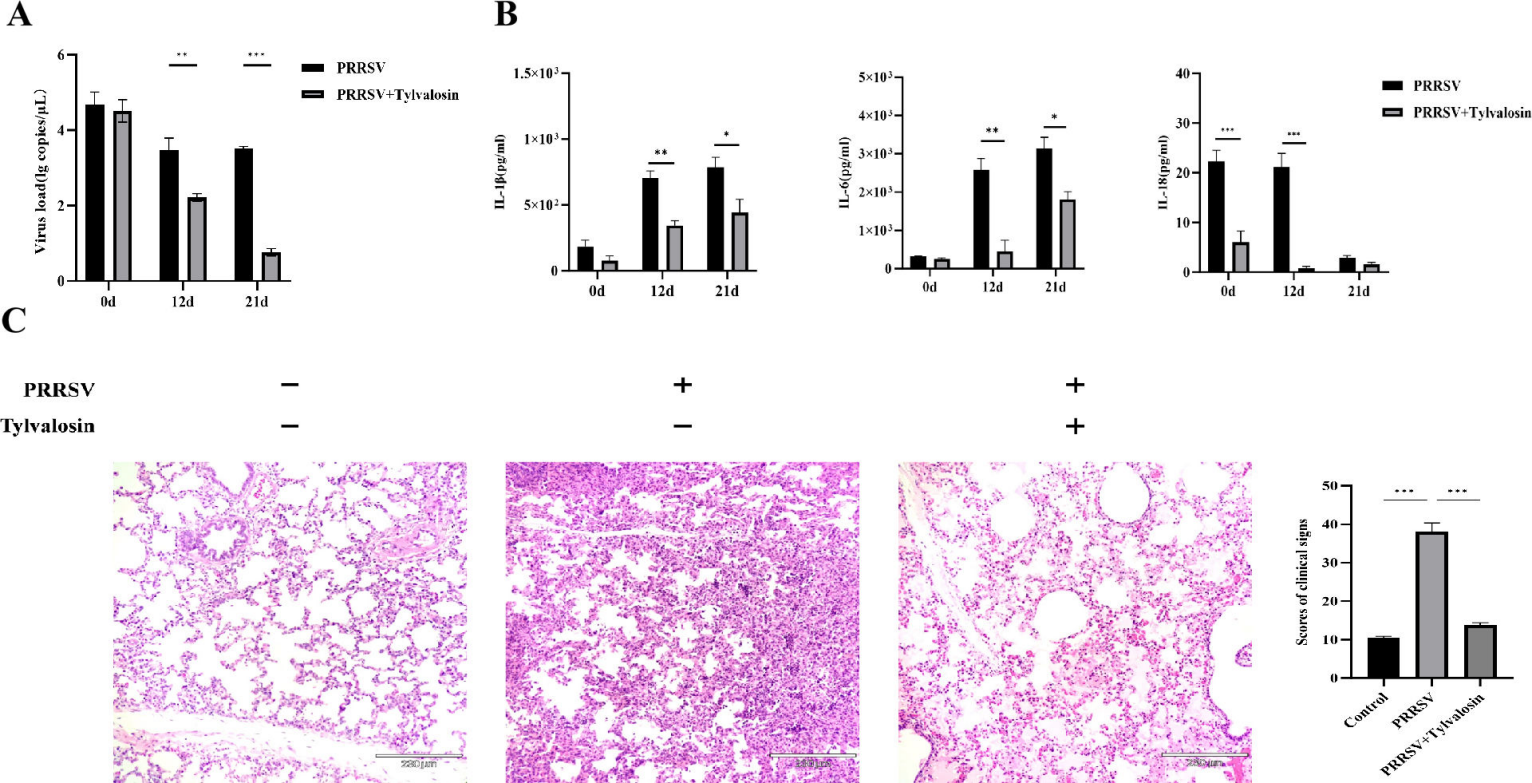
Tylvalosin tartrate alleviates clinical symptoms and suppresses viral replication in piglets infected with lineage 1 (L1) PRRSV. (**A**) Serum viral RNA load (log10 copies/µL) in naturally L1 PRRSV-infected piglets from a clinical trial, measured by RT-qPCR. Piglets were either untreated or treated with tylvalosin tartrate (1,000 mg/kg in feed) for 14 days. Data are shown at days 0, 12, and 21. By day 21, the viral RNA in the treated group had significantly decreased compared to that in the untreated group. (**B**) Serum levels of inflammatory cytokines (IL-1β, IL-6, and IL-18) in the same piglets from panel A, measured by ELISA at the indicated time points. A distinct kinetic profile was observed for IL-18, which showed an initial peak followed by a declining trend in the control group, whereas tylvalosin tartrate treatment maintained its suppression. (**C**) Representative images of hematoxylin and eosin (H&E)-stained lung tissue sections (left panel) and quantitative lung lesion scores (right panel) from experimentally infected piglets. Groups: Mock-infected, L1 PRRSV (TJ-C6)-infected, and L1 PRRSV-infected + tylvalosin tartrate-treated (1,000 mg/kg in feed from day 5 post-infection). Scale bar = 100 µm. Data are presented as mean ± SEM. *P < 0.05, **P < 0.01, and ***P < 0.001.

To evaluate tylvalosin tartrate’s anti-PRRSV efficacy, 20 piglets naturally infected with L1 PRRSV and testing negative for other major pathogens, such as African swine fever virus (ASFV), pseudorabies virus (PRV), and classical swine fever virus (CSFV), via PCR testing were chosen. These selected piglets were divided into a treated group (administered at 1,000 mg/kg in feed for 14 days) and an untreated group. Initially, there was no difference in the serum viral RNA, which was quantified as log10 copies value by real-time fluorescence quantitative PCR (RT-qPCR). Nevertheless, by day 12, it was remarkably lower in the treated group. By day 21, the viral RNA in the treated group had significantly decreased compared to that in the untreated group (Fig. 1A). Meanwhile, the inflammatory cytokines (IL-1β, IL-6, and IL-18) had significantly declined by day 12 post-treatment. A distinct kinetic profile was observed for IL-18, which showed an initial peak followed by a declining trend in the control group, whereas tylvalosin tartrate treatment maintained its suppression (Fig. 1B).

To further evaluate the protective efficacy of tylvalosin tartrate treatment against lung lesions caused by L1 PRRSV, piglets under experimental conditions were employed (29, 30). After tylvalosin tartrate treatment, the alveolar septa displayed better integrity and distinctness, the alveolar structure improved, and the areas of lung tissue congestion were notably reduced compared with those in the infected control group (Fig. 1C). The lung lesion scores of the tylvalosin tartrate-treated group were significantly lower than those of the infected control group (Fig. 1C), suggesting that tylvalosin tartrate is effective in alleviating PRRSV-induced lung tissue damage. These findings further emphasized the significant protective effect of tylvalosin tartrate against PRRSV infection.

### Tylvalosin tartrate inhibits the replication of L1 and other major PRRSV lineages in PAMs

**Fig 2:**
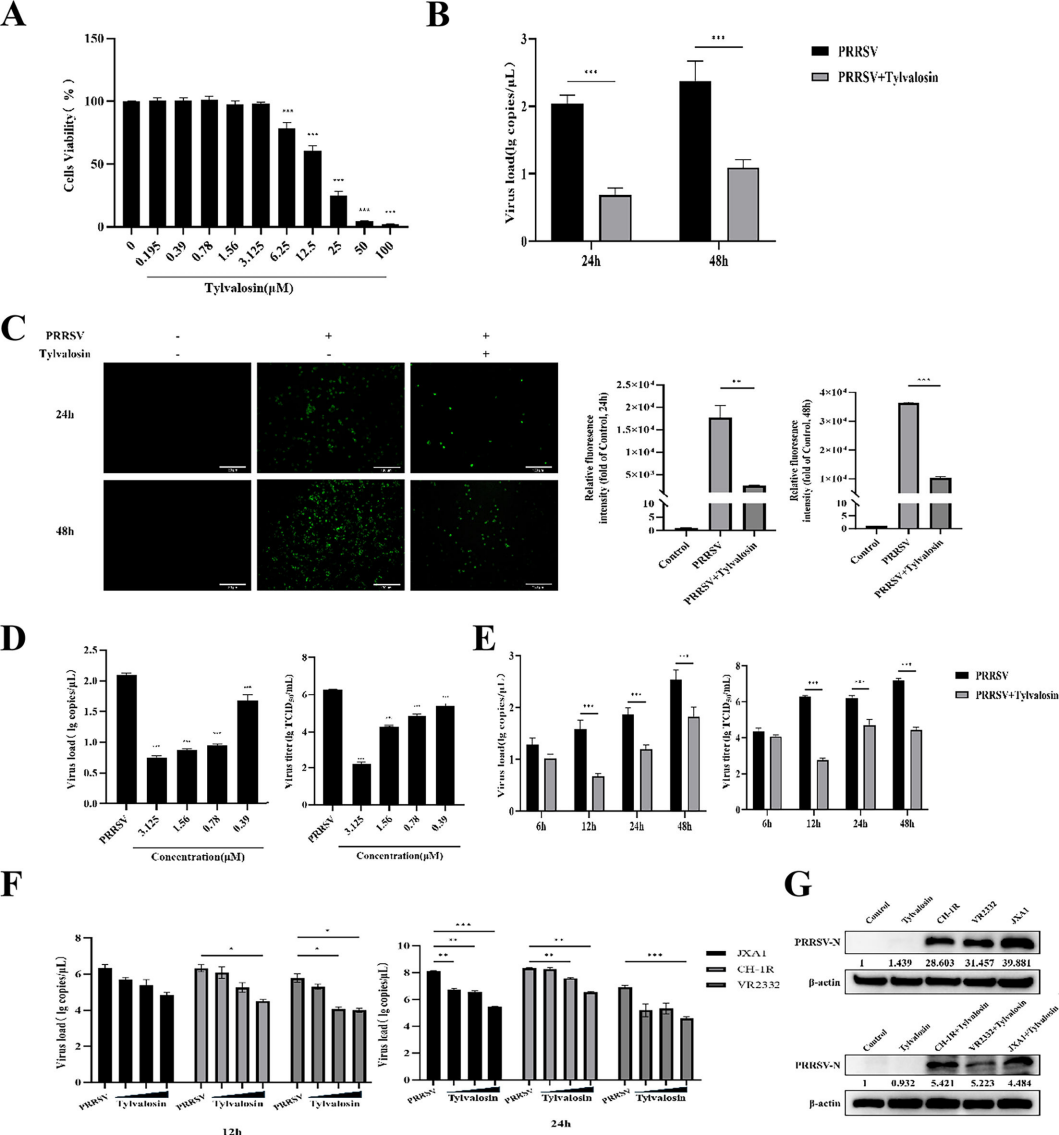
Tylvalosin tartrate broadly inhibits the replication of multiple PRRSV lineages in porcine alveolar macrophages (PAMs) *in vitro*. (**A**) Cytotoxicity assessment of tylvalosin tartrate in PAMs using CCK-8 assay. (**B, C**) Inhibition of L1 PRRSV (strain TJ-C6) replication by post-infection treatment with 3.125 µM tylvalosin tartrate. (**B**) Viral mRNA copies measured by qPCR at 24 and 48 h post-infection (hpi). (**C**) Representative immunofluorescence assay (IFA) images showing PRRSV N protein (green) at 48 hpi. Nuclei are stained with DAPI (blue). Quantitative analysis of fluorescence intensity. Data are presented as the mean integrated fluorescence density (or mean fluorescence intensity) from at least three independent experiments. Scale bar = 100 µm. (**D, E**) Dose-dependent (**D**) and time-course (**E**) effects of tylvalosin tartrate on TJ-C6 replication, assessed by qPCR. (**F, G**) Broad-spectrum antiviral activity of tylvalosin tartrate (3.125 µM). (**F**) Viral mRNA copies of representative strains from lineages 8.1 (CH-1a-like), 5 (VR2332-like), and 8.3 (JXA1-like) measured by qPCR at 12 or 24 hpi. (**G**) Western blot analysis of PRRSV N protein expression for the indicated strains at 24 hpi. Data are presented as mean ± SEM. *P < 0.05, **P < 0.01, and ***P < 0.001.

To confirm the anti-PRRSV efficacy of tylvalosin tartrate *in vitro*, cytotoxicity assessment determined the maximum safe concentration of tylvalosin tartrate for PAMs as 3.125 µM (Fig. 2A). Given that tylvalosin tartrate is primarily used for post-onset treatment in clinical settings, the post-infection treatment approach was adopted for antiviral research in this study. After 1 h of viral adsorption, post-infection treatment with 3.125 µM tylvalosin tartrate notably diminished the replication of the L1 strain TJ-C6 (GenBank no. PQ273406), as demonstrated by the reduced viral mRNA copies detected via qPCR (Fig. 2B) and the specific green fluorescence observed with immunofluorescence assay (IFA) (Fig. 2C) at 24 and 48 h post-infection (hpi). Dose- and time-course experiments identified 3.125 µM at 12 hpi as optimal for inhibiting TJ-C6 replication (Fig. 2D and E).

To further evaluate the breadth of antiviral activity of tylvalosin tartrate, other major prevalent PRRSV-2 strains in China, such as CH-1R-like (lineage 8.1), VR2332-like (lineage 5), and JXA1-like (lineage 8.3), were also selected. At the safe concentration of 3.125 µM, tylvalosin tartrate significantly inhibited the replication of all tested strains, reducing viral mRNA copies (Fig. 2F) and N protein expression (Fig. 2G) at 12 hpi or 24 hpi. These results demonstrate that tylvalosin tartrate possesses broad antiviral activity against multiple PRRSV lineages *in vitro*.

### Transcriptomic analysis links the antiviral effect of tylvalosin tartrate to pyroptosis

**Fig 3:**
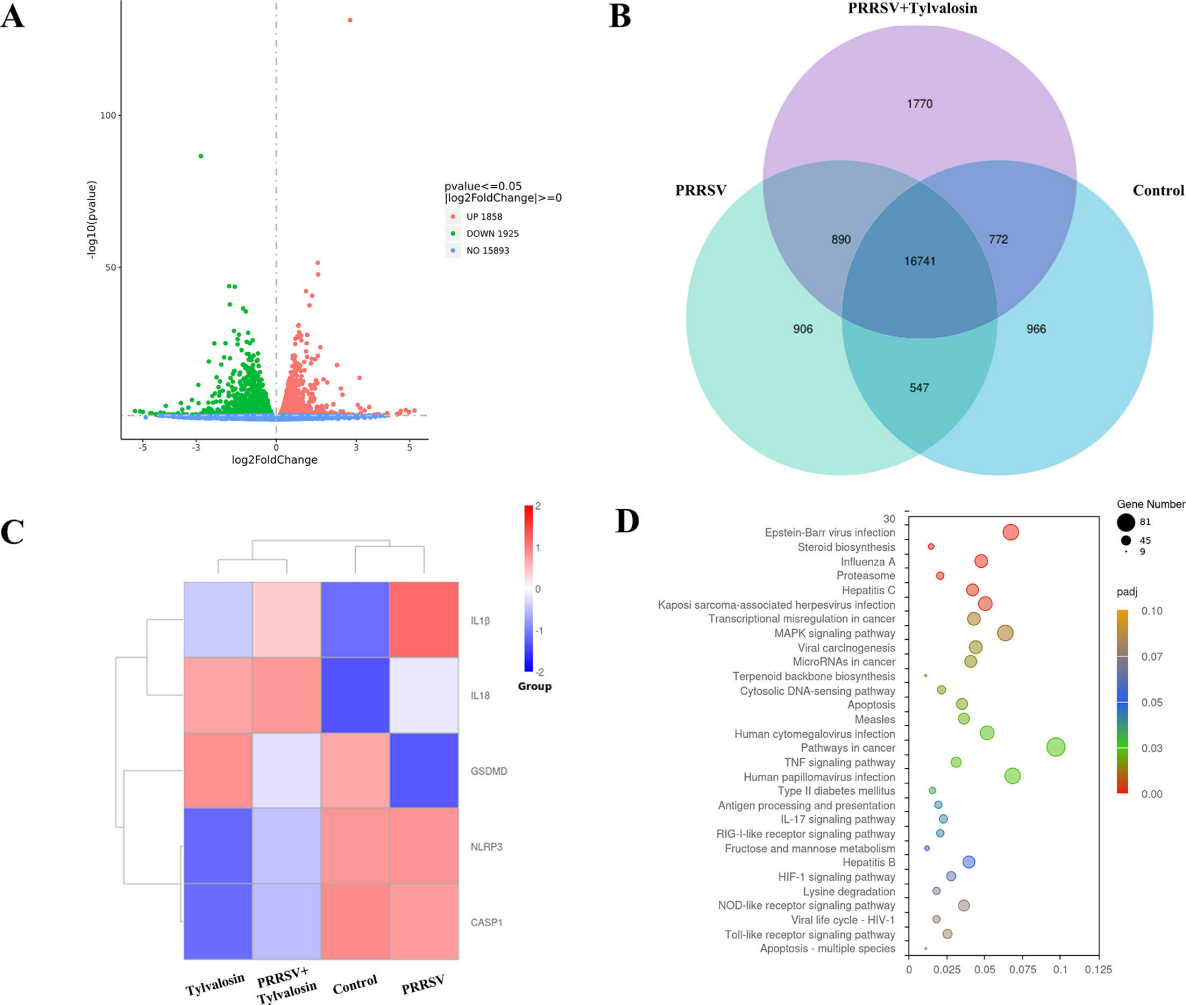
Transcriptomic analysis links the antiviral effect of tylvalosin tartrate to the suppression of pyroptosis-related pathways. PAMs were treated as follows: control (Mock), L1 PRRSV (TJ-C6)-infected, and L1 PRRSV-infected + 3.125 µM tylvalosin tartrate. RNA was harvested at 12 hpi for sequencing. (**A**) Volcano plot showing differentially expressed genes (DEGs) between the tylvalosin tartrate-treated infected group and the infected control group. (**B**) Venn diagram illustrating the numbers of unique and shared DEGs among the three comparison groups. (**C**) Heatmap depicting the expression levels of key pyroptosis-related genes (NLRP3, CASP1, GSDMD, IL-1β, and IL-18) across the three treatment groups. (**D**) KEGG pathway enrichment analysis of downregulated DEGs in the tylvalosin tartrate-treated infected group compared to the infected control.

To initially investigate the anti-L1 PRRSV mechanism of tylvalosin tartrate, PAMs from different groups (control, TJ-C6 infected, and TJ-C6+tylvalosin tartrate) were harvested at 12 hpi for transcriptomic analysis. RNA-Seq revealed 1,858 upregulated and 1,925 downregulated genes in the tylvalosin-treated infected group (Fig. 3A). Venn diagram analysis showed unique and shared differentially expressed genes (DEGs) (Fig. 3B). Expression of key pyroptosis-related genes (e.g., NLRP3, CASP1, GSDMD, IL-1β, and IL-18) was markedly reduced by tylvalosin tartrate treatment (Fig. 3C). KEGG enrichment analysis confirmed that the downregulated DEGs were significantly enriched in pathways directly associated with pyroptosis, most notably the “NOD-like receptor signaling pathway” and the “Pyroptosis” pathway (Fig. 3D), suggesting tylvalosin tartrate mitigates PRRSV-induced pyroptosis.

### Tylvalosin tartrate reduces L1 PRRSV-induced cellular pyroptosis in PAMs

**Fig 4:**
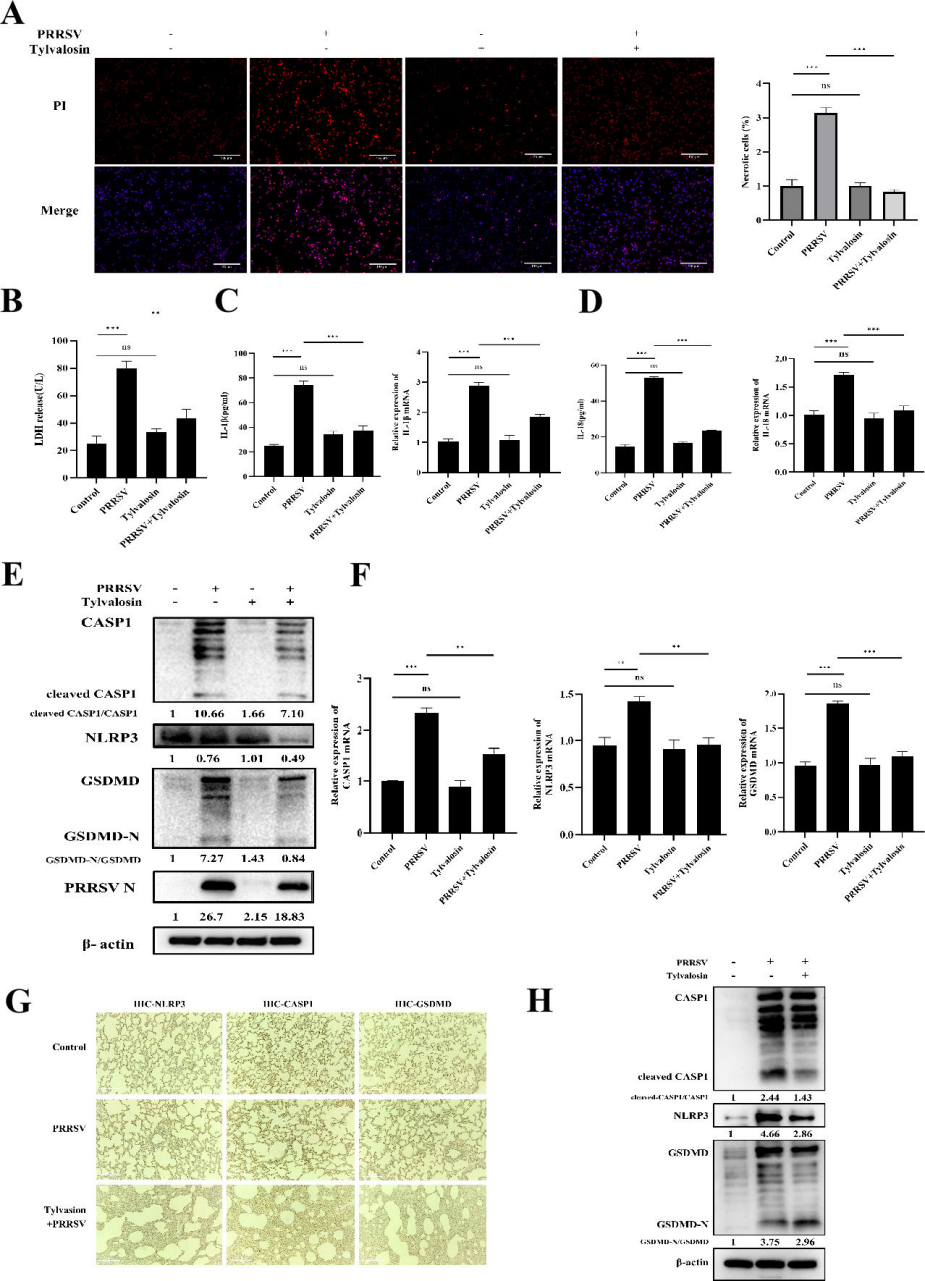
L1 PRRSV infection induces cellular pyroptosis in PAMs and piglet lungs, which is inhibited by tylvalosin tartrate. PAMs were infected with TJ-C6 (multiplicity of infection [MOI] =1 ) and treated with or without 3.125 µM tylvalosin tartrate. Analyses were performed at 12 hpi, unless specified. (**A**) Representative images of propidium iodide (PI) and DAPI staining. PI-positive (purple) nuclei indicate cells undergoing pyroptosis. Nuclei were stained with DAPI (blue). Cells undergoing pyroptosis with permeabilized membranes are stained by propidium iodide (PI, red); colocalization with DAPI appears purple. Scale bar = 100 µm. The bar graph shows the quantification of PI-positive cells. (**B**) Lactate dehydrogenase (LDH) release assay. (**C, D**) mRNA expression (**C**) and protein secretion (**D**) of mature IL-1β and IL-18. (**E, F**) mRNA expression (**E**) and protein levels (**F**) of key pyroptosis components (NLRP3, cleaved CASP1, and GSDMD-N terminus). β-actin served as a loading control. (**G, H**) *In vivo* validation using lung tissues from experimentally infected piglets (as in Fig. 1C). (**G**) Immunohistochemistry (IHC) staining for GSDMD-N protein. Scale bar = 100 µm. (**H**) Western blot analysis of cleaved CASP1 and GSDMD-N protein levels in lung tissues. Data are presented as mean ± SEM. *P < 0.05, **P < 0.01, and ***P < 0.001; n.s., not significant.

To investigate whether L1 PRRSV could initiate cellular pyroptosis, PAMs were inoculated with TJ-C6 *in vitro* and harvested at 12 hpi. The results demonstrated that TJ-C6 infection triggered pyroptosis, as evidenced by the following: (i) an increase in propidium iodide (PI)-positive nuclei, which appeared purple, implying that the cells with permeabilized membranes were experiencing pyroptosis and the rate of necrotic cell death was significantly higher than that in the mock-infected group (Fig. 4A); (ii) an elevation in lactate dehydrogenase (LDH) release (Fig. 4B); (iii) a rise in the mRNA and protein levels of IL-1β and IL-18 (Fig. 4C and D); (iv) an upregulation of the mRNA and protein levels of NLRP3, cleaved CASP1, and the N-terminal pore-forming fragment of GSDMD (GSDMD-N) (Fig. 4E). The cleaved, active forms of CASP1 and GSDMD-N are both crucial for the induction of pyroptotic cell death. Densitometric analysis confirmed significant increases. Treatment with tylvalosin tartrate (3.125 µM) significantly reduced all pyroptosis markers (Fig. 4A through F). Notably, tylvalosin tartrate alone did not induce LDH release or elevate cytokine levels, excluding nonspecific cytotoxicity. Meanwhile, immunohistochemistry (IHC) and western blotting analysis of lung tissues from infected piglets in *vivo* revealed an increase in pyroptosis-related protein-positive cells within inflammatory regions, along with the upregulated expression of CASP1 and GSDMD-N proteins (Fig. 4G and H). These findings were consistent with the *in vitro* results, further validating that the infection triggers pyroptosis in lung tissue. Moreover, treatment with tylvalosin tartrate (1,000 mg/kg) in infected piglets led to a significant reduction in the number of pyroptosis-related protein-positive cells and downregulated the expression levels of pyroptosis-related protein markers in lung tissues (Fig. 4G and H), indicating that tylvalosin tartrate can also effectively inhibit pyroptosis *in vivo*.

**Fig 5:**
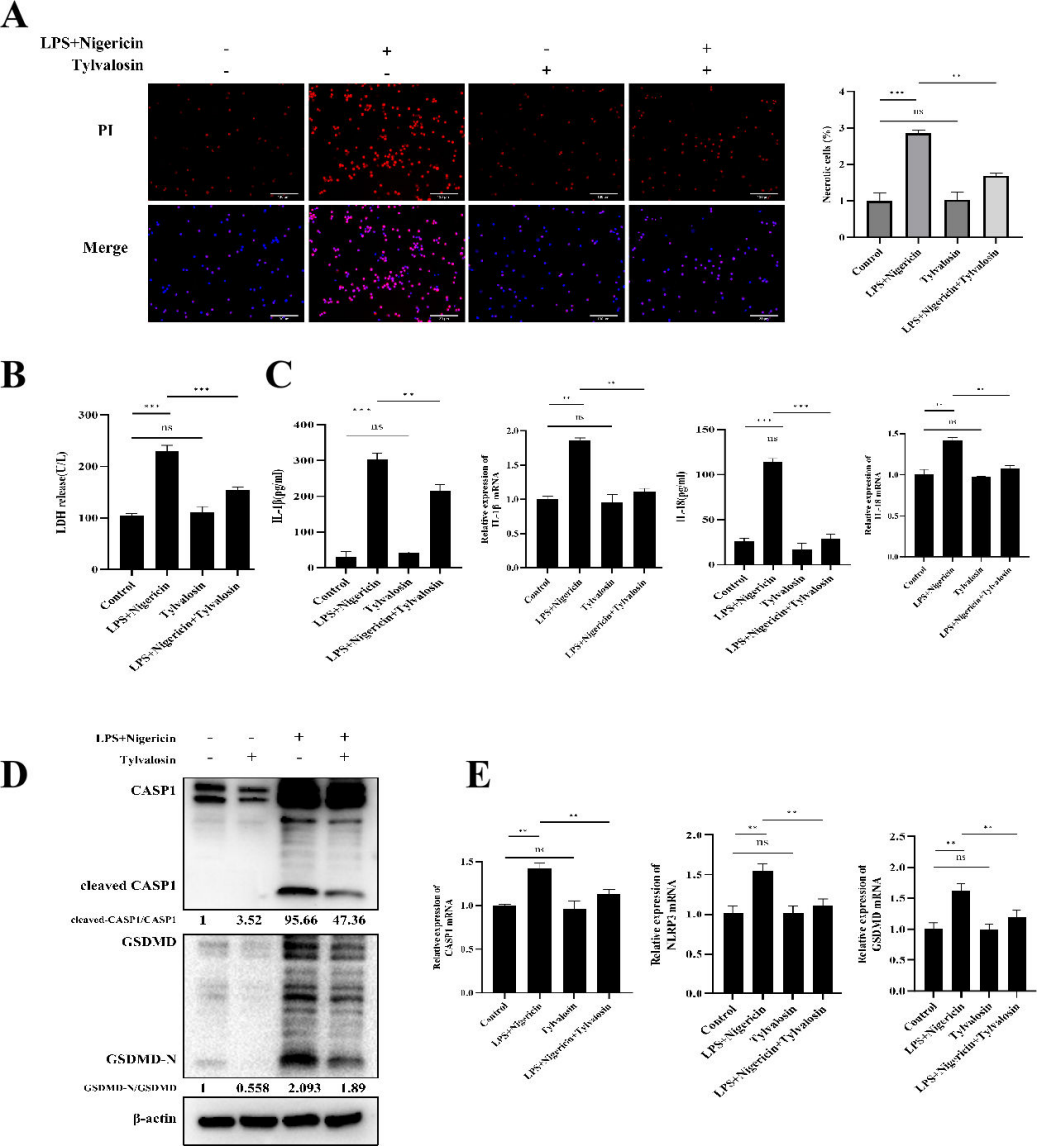
Tylvalosin tartrate directly inhibits LPS/nigericin-induced pyroptosis in PAMs. PAMs were primed with LPS and then stimulated with nigericin to induce pyroptosis, followed by treatment with or without 3.125 µM tylvalosin tartrate. (**A**) Representative images of PI and DAPI staining and quantification of PI-positive cells. Scale bar = 100 µm. (**B**) LDH release assay. (**C**) Secretion levels and mRNA expression of mature IL-1β and IL-18 in cell culture supernatants. (**D, E**) Protein levels (**D**) and mRNA expression (**E**) of NLRP3, cleaved CASP1, and GSDMD-N. Data are presented as mean ± SEM. *P < 0.05, **P < 0.01, and ***P < 0.001; n.s., not significant.

To exclude indirect effects mediated by viral inhibition, pyroptosis was induced in PAMs using lipopolysaccharide (LPS) and nigericin. In this *in vitro* model, the cells were subsequently treated with tylvalosin tartrate, and its effects on pyroptosis were evaluated by measuring PI staining, LDH release, activation of pyroptosis-related proteins, and secretion of IL-1β and IL-18. Tylvalosin tartrate similarly reduced PI staining (Fig. 5A), LDH release (Fig. 5B), IL-1β/IL-18 secretion and mRNA expression (Fig. 5C), as well as mRNA expression and protein levels of NLRP3, cleaved CASP1, and GSDMD-N (Fig. 5D and E) in this model, confirming a direct inhibitory effect on the pyroptosis pathway.

### Tylvalosin tartrate inhibits L1 PRRSV replication by suppressing cellular pyroptosis

**Fig 6:**
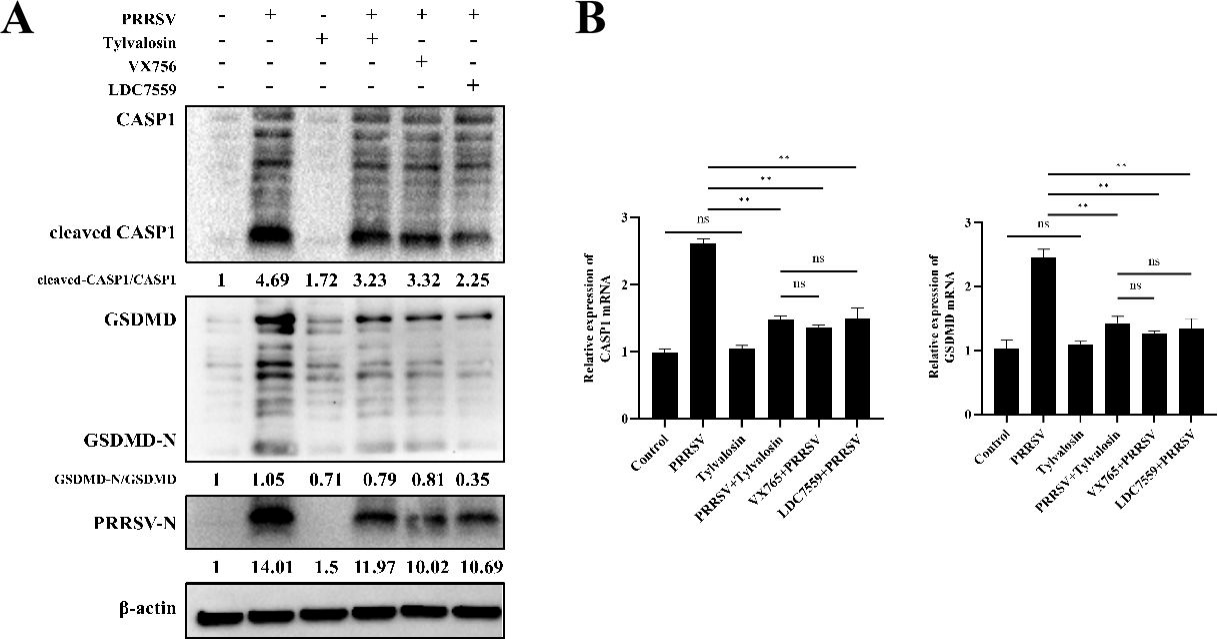
Inhibition of pyroptosis key executors caspase-1 or GSDMD suppresses PRRSV replication. PAMs were pretreated with the CASP1 inhibitor VX765, the GSDMD inhibitor LDC7559, or 3.125 µM tylvalosin tartrate, followed by L1 PRRSV (TJ-C6) infection. (**A, B**) Western blot analysis (**A**) and corresponding densitometric quantification (**B**) showing the protein levels of cleaved CASP1, GSDMD-N, and PRRSV N protein. Data are presented as mean ± SEM. *P < 0.05, **P < 0.01, and ***P < 0.001; n.s., not significant.

To explore the relationship between tylvalosin tartrate, key proteins of cellular pyroptosis (specifically CASP1 and GSDMD), and PRRSV replication, we assessed whether CASP1 or GSDMD was required for PRRSV replication. Our investigation revealed that inhibition of key pyroptosis proteins hindered PRRSV replication. Pretreatment with the CASP1 inhibitor VX765 or the GSDMD inhibitor LDC7559 downregulated the expression levels of pyroptosis-related protein markers (cleaved-CASP1 and GSDMD-N) (Fig. 6A and B); notably, both inhibitors significantly reduced PRRSV N protein (Fig. 6A). Tylvalosin tartrate treatment mirrored these effects (Fig. 6A and B), indicating it suppresses PRRSV replication by inhibiting CASP1-/ GSDMD-mediated pyroptosis.

### Tylvalosin tartrate inhibits pyroptosis via the TLR4/NF-κB pathway

**Fig 7:**
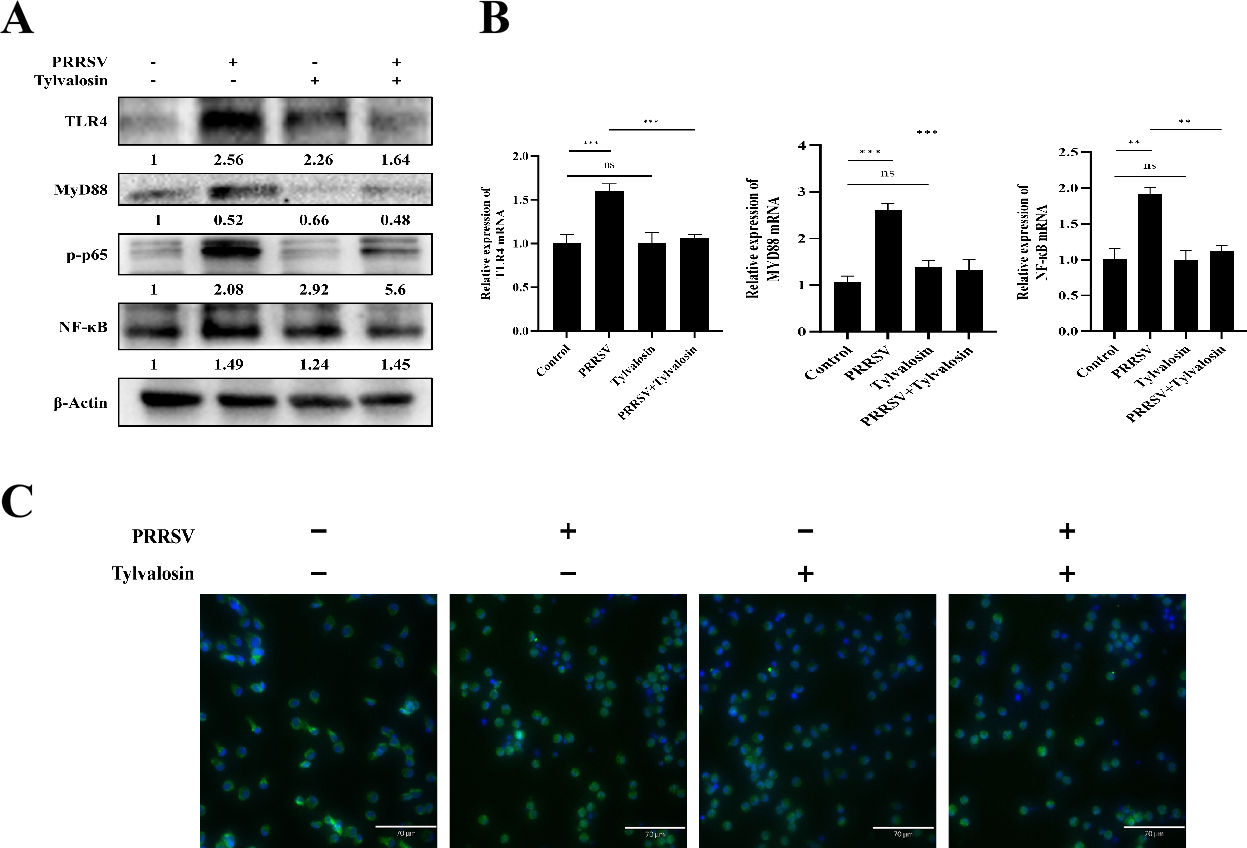
Tylvalosin tartrate inhibits the activation of the TLR4/NF-κB signaling pathway induced by L1 PRRSV infection. PAMs were infected with TJ-C6 and treated with or without 3.125 µM tylvalosin tartrate. (**A**) mRNA expression levels of TLR4 and NF-κB p65 at 12 hpi. (**B**) Western blot analysis of TLR4, total p65, phosphorylated p65 (P-P65), and nuclear p65 protein levels. β-actin and lamin B1 served as cytoplasmic and nuclear loading controls, respectively. (**C**) Representative immunofluorescence images showing the cellular localization of NF-κB p65 (red). Nuclei are stained with DAPI (blue). The localization of p65 (green) and the cytosolic marker DAPI (blue) was visualized through indirect IFA. Scale bar = 25 µm. Data are presented as mean ± SEM. *P < 0.05, **P < 0.01, and ***P < 0.001; n.s., not significant.

Since TLR4/NF-κB signaling promotes pyroptosis during PRRSV infection (11), we examined the impact of tylvalosin tartrate. Infection with TJ-C6 elevated the mRNA and protein levels of TLR4 and triggered the phosphorylation and nuclear translocation of NF-κB p65. Conversely, treatment with tylvalosin tartrate markedly attenuated these responses (Fig. 7A and B). Immunofluorescence assays further confirmed the diminished nuclear translocation of p65 (Fig. 7C).

**Fig 8:**
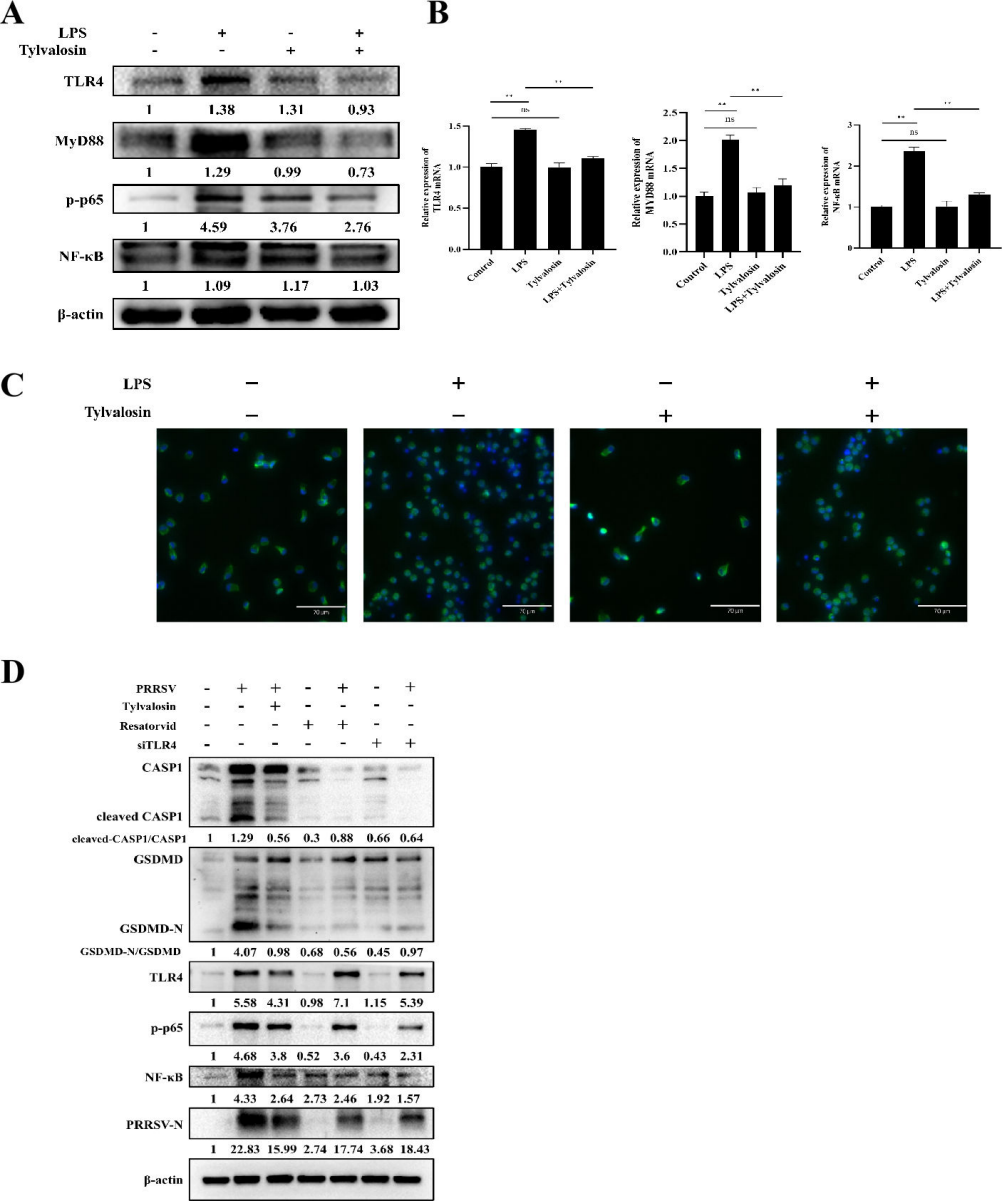
Pharmacological inhibition or genetic knockdown of TLR4 replicates the antiviral and anti-pyroptotic effects of tylvalosin tartrate. (**A–C**) PAMs were stimulated with LPS to activate TLR4 and treated with or without tylvalosin tartrate. (**A**) Western blot of TLR4 and p-p65 levels. (**B**) Quantification of TLR4, MYD88 and NF-κB by western blot. (**C**) Immunofluorescence images of p65 localization. Immunofluorescence analysis of p65 (green) nuclear translocation. Nuclei were stained with DAPI (blue): scale bar = 25 µm. (**D**) PAMs were pretreated with the TLR4-specific inhibitor resatorvid or transfected with TLR4-targeting siRNA, followed by PRRSV infection. Western blot analysis of TLR4, p-p65, pyroptosis-related proteins (cleaved CASP1 and GSDMD-N), and PRRSV N protein. Data are presented as mean ± SEM. *P < 0.05, **P < 0.01, and ***P < 0.001; n.s., not significant.

To rule out that TLR4 inhibition was secondary to reduced viral replication, LPS was used to activate TLR4. Tylvalosin tartrate prevented LPS-induced TLR4 degradation, reduced phospho-p65 levels, and inhibited p65 nuclear translocation (Fig. 8A through C). Furthermore, pretreatment with the TLR4 inhibitor resatorvid reduced expression of TLR4, phospho-p65, pyroptosis-related proteins, and PRRSV-N, similar to tylvalosin tartrate (Fig. 8D). Additional validation using TLR4-specific siRNA showed that TLR4 knockdown reduced PRRSV-induced pyroptosis and viral replication (Fig. 8D). These results indicate that tylvalosin tartrate inhibits PRRSV-induced pyroptosis primarily through the TLR4/NF-κB pathway.

## DISCUSSION

Tylvalosin tartrate, a third-generation macrolide derivative, has been empirically associated with improved outcomes in PRRSV-affected herds (31), yet its precise antiviral mechanism and spectrum of activity have remained unclear. This study not only confirms its efficacy against the contemporary epidemic lineage 1 PRRSV both in *vivo* and *in vitro* but also critically expands its known antiviral range. We demonstrate that tylvalosin tartrate significantly inhibits the replication of other major co-circulating strains, including CH-1a (lineage 8.1), VR2332 (lineage 5), and JXA1 (lineage 8.3), in PAMs. This broad-spectrum activity against genetically diverse PRRSV isolates positions tylvalosin tartrate as a promising host-directed therapeutic candidate.

The translational relevance of our *in vitro* findings is strongly supported by pharmacokinetic bridging. The effective in *vitro* concentration (3.125 µM) aligns with estimated plasma levels (~2-5 µM) (32) achieved by the clinical oral dose (1,000 mg/kg). The more pronounced viral clearance observed *in vivo*, compared to the partial inhibition *in vitro*, likely stems from the compound’s combined direct antiviral and systemic immunomodulatory effects within the intact host, a complexity not recapitulated in cell culture models.

Pyroptosis is a programmed cell death executed by GSDMD, characterized by membrane pore formation, LDH/cytokine release, and PI uptake (33–36). A central finding of this work is the identification of a novel host-directed mechanism. We established that L1 PRRSV infection robustly induces GSDMD-executed pyroptosis in PAMs and piglet lungs. Tylvalosin tartrate treatment markedly reduced all associated hallmarks, including PI uptake, LDH release, and the maturation of IL-1β/IL-18. Crucially, the pharmacological inhibition of key pyroptosis executors (CASP1 or GSDMD) directly impaired PRRSV replication, mirroring the effect of tylvalosin tartrate. This confirms that the drug’s antiviral action is mediated, at least in part, through the suppression of this lytic cell death pathway. Importantly, these effects were not attributable to nonspecific cytotoxicity.

**Fig 9:**
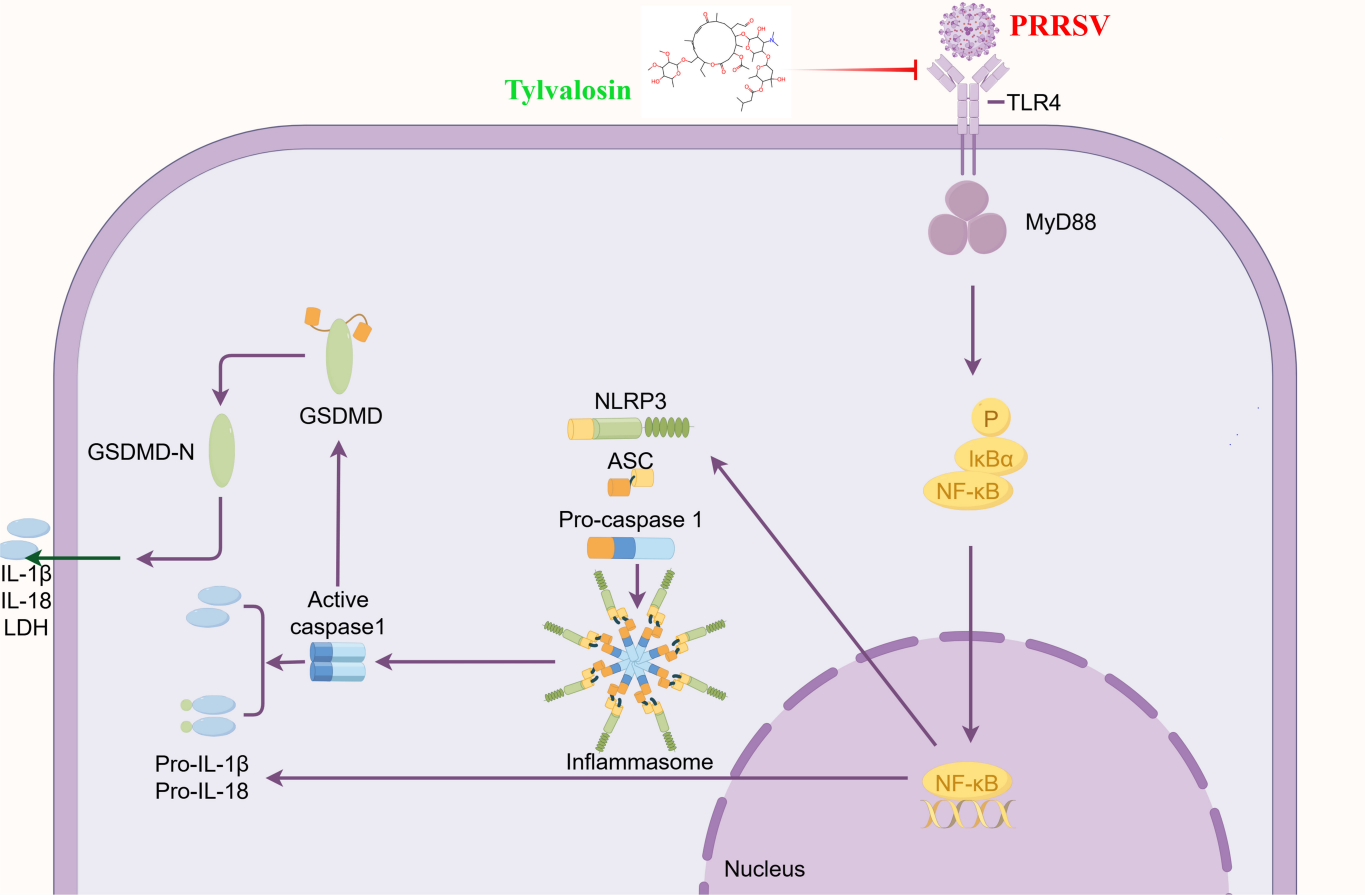
Schematic diagram illustrating the proposed mechanism by which tylvalosin tartrate inhibits PRRSV replication. The graphic summarizes the main findings: PRRSV infection activates the host TLR4/NF-κB signaling pathway, leading to the transcriptional upregulation of inflammasome components (e.g., NLRP3 and pro-IL-1β) and gasdermin D (GSDMD). This promotes NLRP3 inflammasome assembly, caspase-1 (CASP1) activation, and subsequent cleavage of GSDMD and pro-IL-1β. Cleaved GSDMD-N forms pores in the cell membrane, inducing pyroptotic cell death and the release of mature IL-1β/IL-18, which may facilitate viral spread and inflammation. Tylvalosin tartrate acts upstream by inhibiting the activation of the TLR4/NF-κB pathway, thereby suppressing the entire cascade of pyroptosis and inflammatory cytokine production, ultimately restricting PRRSV replication across multiple viral lineages.

TLR4, activated by pathogens or damage signals, initiates inflammation and can promote viral replication (37, 38). PRRSV-induced IL-1β secretion is TLR4-dependent (39). Additionally, NF-κB, downstream of TLR4, is a key regulator of inflammatory responses and a target for viruses like PRRSV (40–42). We further delineated the upstream signaling events targeted by tylvalosin tartrate. PRRSV infection activated the TLR4/NF-κB axis, a key driver of inflammatory gene expression. Tylvalosin tartrate effectively suppressed TLR4 upregulation, NF-κB p65 phosphorylation, and its nuclear translocation. The functional centrality of this pathway was unequivocally demonstrated through both pharmacological inhibition (resatorvid) and genetic knockdown (siRNA) of TLR4, which replicated the dual anti-pyroptotic and antiviral effects of the drug. The profound suppression of CASP1 activation by resatorvid underscores TLR4’s role as a master upstream regulator essential for priming the pyroptotic response to PRRSV. Therefore, tylvalosin tartrate exerts its function by modulating the TLR4/NF-κB/pyroptosis axis. A schematic summary is shown in Fig. 9.

The cytokine kinetics in infected piglets revealed a distinct pattern for IL-18, which displayed an early peak followed by a decline in untreated animals, unlike the sustained elevation of IL-1β and IL-6. This distinct kinetic profile may reflect differential regulation of these cytokines. While both IL-1β and IL-18 are produced upon inflammasome activation, their secretion and subsequent regulation can be subject to different checkpoints. The early IL-18 surge could indicate an initial, rapid inflammatory response that is subsequently downregulated, whereas the persistent elevation of IL-1β and IL-6 suggests a sustained inflammatory state during established infection. Notably, tylvalosin tartrate effectively suppressed all three cytokines, including the early IL-18 surge, highlighting its capacity to modulate complex and dynamic inflammatory responses in *vivo*. Additionally, the observed broad-spectrum activity across distinct viral lineages can be parsimoniously explained by this host-targeted mechanism. PRRSV strains likely converge on hijacking conserved host pro-inflammatory pathways, such as TLR4/NF-κB, to facilitate their replication. By disrupting this common host interface, tylvalosin tartrate achieves lineage-independent antiviral efficacy. While our detailed mechanistic dissection was performed with an L1 strain, the consistent inhibition of other lineages strongly suggests a conserved mode of action, offering a strategic advantage for controlling a heterogeneous viral population.

In conclusion, this study elucidates a novel mechanism by which tylvalosin tartrate restricts PRRSV replication: it targets the host’s TLR4/NF-κB signaling pathway to suppress the subsequent GSDMD-dependent pyroptosis. This “old drug, new use” strategy, which counters both viral replication and excessive inflammation, provides a robust scientific rationale for its clinical application. Future research confirming the precise modulation of this axis by tylvalosin tartrate during infection by other PRRSV lineages will further solidify this model and inform its optimal use in PRRS management.

## MATERIALS AND METHODS

### Animals

Piglets free of PRRSV, aged 4 weeks, were procured from SPF (Beijing) Biotechnology Co., Ltd.

### Cells and virus

Porcine alveolar macrophages (PAMs) were isolated from 3-week-old piglets confirmed to be free of PRRSV antigen and antibody, as previously described with modifications (5, 6). Briefly, the lungs were aseptically excised, and pre-cooled sterile PBS (containing antibiotics) was instilled through the trachea for bronchoalveolar lavage. The lavage fluid was collected and centrifuged at 500 × *g* for 10 min to obtain the cell pellet. The cells were resuspended and washed twice with PBS and then seeded in culture dishes. They were cultured in RPMI-1640 medium supplemented with 10% fetal bovine serum at 37°C and 5% CO2_2_ for 2–4 h to allow adherence. Non-adherent cells were subsequently washed away, and the adherent cells obtained were identified as PAMs. The following PRRSV-2 strains were used: L1 strain TJ-C6 (GenBank no. PQ273406.1), lineage 8.1 strain CH-1a (GenBank no. AF066183), lineage 5 strain VR2332 (GenBank no. AF046835‌), and lineage 8.3 strain JXA1 (GenBank no. EF112445).

### Clinical and experimental animal experiments

For the clinical trial, 20 L1 PRRSV-positive piglets were divided into two groups: the treated group, which received 1,000 mg/kg of a 20% tylvalosin tartrate premix produced by ECO-BIOK in their feed for 14 days, and the untreated group. Serum was collected for viremia (RT-qPCR) and cytokine (ELISA) analysis. For experimental infection, 15 PRRSV-naïve piglets were divided into three groups (*n* = 5): mock-infected, TJ-C6-infected (via intranasal inoculation), and TJ-C6-infected + tylvalosin tartrate (1,000 mg/kg feed from day 5 post-infection). After 3 weeks, lungs were collected for histopathology and lesion scoring based on established criteria (42). Briefly, gross lung lesions were graded according to the percentage of the lung surface affected by pneumonia. Histopathological lesions in H&E-stained sections were scored on a 0–3 scale based on the severity of interstitial pneumonia: 0 = no lesion, 1 = mild, 2 = moderate/multifocal, and 3 = severe.

### Cytotoxicity and antiviral activity assays

Cytotoxicity was assessed via CCK-8. For antiviral assays, PAMs were infected with different PRRSV strains (MOI = 1). After 1 h of viral adsorption, the inoculum was removed, cells were washed with PBS, and then cultured in fresh medium containing 3.125 µM tylvalosin tartrate for the indicated durations. For all assays involving tylvalosin tartrate treatment, a control group treated with the drug alone (at 3.125 µM) was included to assess any effects independent of viral infection or stimulation. Viral RNA (RT-qPCR), titer (TCID_50_), and N protein (IFA) were assessed at indicated time points.

### Real-time RT-PCR

Viral load was quantified by RT-qPCR using a standard curve generated from a serial dilution of a plasmid with known copy numbers containing the PRRSV ORF7 gene. Results are expressed as log_10_ genomic copies per microliter (log_10_ copies/µL) of serum. Samples with Ct values exceeding the cycle threshold of the lowest standard were considered below the limit of detection (LOD).

### Transcriptomic sequencing

Total RNA from PAMs (control, TJ-C6, and TJ-C6 + tylvalosin tartrate) at 12 hpi was subjected to RNA-Seq. DEGs and KEGG pathways were analyzed.

### Pyroptosis assays

LDH release (cytotoxicity assay kit), cytokine levels (ELISA), PI/DAPI staining, and mRNA/protein levels of pyroptosis markers (qPCR and Western blotting) were performed.

### Inhibition or agitation of signaling pathways

PAMs were pretreated with inhibitors (VX765, LDC7559, and resatorvid) or transfected with siRNA targeting CASP1, GSDMD, or TLR4 prior to infection or LPS/nigericin treatment.

### PI staining

Propidium iodide (PI) staining was used to detect pyroptotic cells. After treatment, cells were incubated with PI (5 µg/mL, Sigma-Aldrich, Cat# P4170) for 15 min at 37°C in the dark. Subsequently, cells were fixed with 4% paraformaldehyde for 15 min, permeabilized with 0.1% Triton X-100, and then stained with DAPI (1 µg/mL, Sigma-Aldrich, Cat# D9542) for 10 min. Images were captured using a fluorescence microscope (Olympus IX83), and the percentage of PI-positive cells was calculated by counting at least five random fields per well.

### RT-qPCR

Total RNA was extracted from cells or lung tissues using TRIzol reagent (Invitrogen, Cat# 15596026) following the manufacturer’s instructions. The concentration and purity of RNA were assessed using a NanoDrop 2000 spectrophotometer. For reverse transcription, 1 µg of total RNA was used to synthesize cDNA with a PrimeScript RT Reagent Kit (Takara, Cat# RR037A). Quantitative real-time PCR (qPCR) was performed using TB Green Premix Ex Taq II (Takara, Cat# RR820A). The cycling conditions were: 95°C for 30 s, followed by 40 cycles of 95°C for 5 s and 60°C for 30 s. The relative expression levels of target genes were calculated using the 2-ΔΔCt method and normalized to the housekeeping gene GAPDH. Primer sequences are available upon request.

### Western blotting

Cells or lung tissue samples were lysed in RIPA buffer (Beyotime, Cat# P0013B) supplemented with protease and phosphatase inhibitors. Protein concentrations were determined using a BCA Protein Assay Kit (Thermo Fisher Scientific, Cat# 23225). Equal amounts of protein (20–30 µg) were separated by SDS-PAGE and then transferred onto PVDF membranes (Millipore, Cat# IPVH00010). The membranes were blocked with 5% non-fat milk in TBST for 1 h at room temperature, followed by overnight incubation at 4°C with specific primary antibodies (see “Antibodies,” below, for details). After washing, the membranes were incubated with horseradish peroxidase (HRP)-conjugated secondary antibodies for 1 h at room temperature. Protein bands were visualized using an enhanced chemiluminescence (ECL) substrate (Thermo Fisher Scientific, Cat# 34580) and imaged with a ChemiDoc MP imaging system (Bio-Rad).

### Immunohistochemistry (IHC)

Lung tissue samples were fixed in 4% paraformaldehyde, embedded in paraffin, and sectioned at 4 µm thickness. Following deparaffinization and rehydration, antigen retrieval was performed by boiling sections in citrate buffer (pH 6.0). Endogenous peroxidase activity was blocked with 3% H_2_O_2_. Sections were then incubated with primary antibodies against GSDMD-N or cleaved CASP1 overnight at 4°C. After washing, sections were incubated with a biotinylated secondary antibody, followed by streptavidin-horseradish peroxidase (HRP). The signal was developed using 3,3'-diaminobenzidine (DAB), and sections were counterstained with hematoxylin. Images were captured under a light microscope (Nikon Eclipse Ci).

### Indirect fluorescent antibody assay (IFA)

PAMs grown on coverslips were fixed with 4% paraformaldehyde for 15 min and permeabilized with 0.1% Triton X-100 for 10 min. After blocking with 1% BSA, cells were incubated with a mouse monoclonal antibody against PRRSV N protein (or NF-κB p65) at 4°C overnight. After washing, cells were incubated with Alexa Fluor 488- or 594-conjugated secondary antibodies (Invitrogen) for 1 h at room temperature. Nuclei were counterstained with DAPI. Coverslips were mounted onto slides with anti-fade mounting medium and examined under a fluorescence microscope (Olympus IX83).

### Antibodies

The following primary antibodies were used in this study: anti-CASP1(D7F10) (rabbit monoclonal, CellSignaling Technology, Cat# 3866, dilution: 1:1,000 for WB); anti-GSDMD(E5O4N) (rabbit monoclonal, CellSignaling Technology, Cat# 69469, dilution: 1:1,000 for WB); anti-TLR4 (rabbit polyclonal, Proteintech, Cat# 19811-1-AP, dilution: 1:1,000 for WB); anti-NF-κB p65 (rabbit monoclonal, Proteintech, Cat# 10745-1-AP, dilution: 1:1,000 for WB); anti-phospho-NF-κBp65 (Ser536) (rabbit monoclonal, Cell Signaling Technology, Cat# 3033, dilution: 1:1,000 for WB); anti-β-actin (mouse monoclonal, Proteintech, Cat# 66009-1-Ig, dilution: 1:20,000 for WB).

### Statistical analysis

Data are presented as mean ± SEM. Statistical significance was determined by one-way ANOVA using GraphPad Prism 8.0. *P < 0.05, **P < 0.01, and ***P < 0.001; n.s., not significant.

## Data Availability

All data needed to evaluate the conclusions in the paper are included in this article.
